# Multitask Learning With Recurrent Neural Networks for Acute Respiratory Distress Syndrome Prediction Using Only Electronic Health Record Data: Model Development and Validation Study

**DOI:** 10.2196/36202

**Published:** 2022-06-15

**Authors:** Carson Lam, Rahul Thapa, Jenish Maharjan, Keyvan Rahmani, Chak Foon Tso, Navan Preet Singh, Satish Casie Chetty, Qingqing Mao

**Affiliations:** 1 Dascena, Inc Houston, TX United States

**Keywords:** deep learning, neural networks, ARDS, health care, multitask learning, clinical decision support, prediction model, COVID-19, electronic health record, risk outcome, respiratory distress, diagnostic criteria, recurrent neural network

## Abstract

**Background:**

Acute respiratory distress syndrome (ARDS) is a condition that is often considered to have broad and subjective diagnostic criteria and is associated with significant mortality and morbidity. Early and accurate prediction of ARDS and related conditions such as hypoxemia and sepsis could allow timely administration of therapies, leading to improved patient outcomes.

**Objective:**

The aim of this study is to perform an exploration of how multilabel classification in the clinical setting can take advantage of the underlying dependencies between ARDS and related conditions to improve early prediction of ARDS in patients.

**Methods:**

The electronic health record data set included 40,703 patient encounters from 7 hospitals from April 20, 2018, to March 17, 2021. A recurrent neural network (RNN) was trained using data from 5 hospitals, and external validation was conducted on data from 2 hospitals. In addition to ARDS, 12 target labels for related conditions such as sepsis, hypoxemia, and COVID-19 were used to train the model to classify a total of 13 outputs. As a comparator, XGBoost models were developed for each of the 13 target labels. Model performance was assessed using the area under the receiver operating characteristic curve. Heat maps to visualize attention scores were generated to provide interpretability to the neural networks. Finally, cluster analysis was performed to identify potential phenotypic subgroups of patients with ARDS.

**Results:**

The single RNN model trained to classify 13 outputs outperformed the individual XGBoost models for ARDS prediction, achieving an area under the receiver operating characteristic curve of 0.842 on the external test sets. Models trained on an increasing number of tasks resulted in improved performance. Earlier prediction of ARDS nearly doubled the rate of in-hospital survival. Cluster analysis revealed distinct ARDS subgroups, some of which had similar mortality rates but different clinical presentations.

**Conclusions:**

The RNN model presented in this paper can be used as an early warning system to stratify patients who are at risk of developing one of the multiple risk outcomes, hence providing practitioners with the means to take early action.

## Introduction

### Background

Acute respiratory distress syndrome (ARDS) is a heterogeneous syndrome broadly characterized by noncardiogenic hypoxia, pulmonary edema, and the need for mechanical ventilation [1,2]. Despite advances made in the diagnosis and management of patients with ARDS, ARDS is present in approximately 10% of the patients admitted to intensive care units (ICUs) worldwide, and mortality is as high as 30% to 40% in most studies [1]. Tools such as the 2016 Kigali modification of the 2012 Berlin criteria have been developed to aid clinicians to diagnose patients with ARDS [3,4]. In addition, the Lung Injury Prediction Score and Early Acute Lung Injury score were developed to identify and stratify patients at risk of developing ARDS based on a collection of physiological variables and predisposing conditions [5-7]. However, ARDS only occurs in a small proportion of patients with a risk factor and currently there is no consensus on how or whether patients should be screened for ARDS. This becomes especially important in the context of patients who are critically ill in the ICU, where health care providers may experience challenges in continuous monitoring and processing large amounts of clinical data from patients. Early warning of impending ARDS should allow implementation of lower tidal volumes in breathing support and more careful fluid management, the 2 main strategies to prevent or reduce the severity of ARDS [8,9].

In the past decade, artificial intelligence has shown great promise in medicine, with potential applications across multiple domains in health care [10]. There have been significant advances in harnessing the power of big data from electronic health records (EHRs) to develop machine learning algorithms to predict the onset of a broad spectrum of medical conditions in patients. A wide variety of such algorithms have been studied and implemented by groups in both academia and industry [11-16]. Previous studies have suggested that the physiological states that exist early in the presentation of ARDS can be used to predict ARDS before the confirmatory tests required by gold standards such as the Berlin criteria [17,18], which requires radiology reports that are by nature subsequent to clinical suspicion. Early prediction is desirable because it would lead to earlier intervention, more time for the careful administration of treatment, or modification of the ongoing treatment, which in turn should lead to improved outcomes such as reduced morbidity and mortality.

### Objectives

In real-world clinical settings, the task is to anticipate multiple diseases or clinical states. In this study, we aimed to demonstrate that multitask learning using deep learning models provides benefits over single-task machine learning models. To this end, we focused on the detection and early prediction of varying severities of ARDS together with sepsis, COVID-19, hypoxemia, and in-hospital mortality. Previous studies have developed multilabel classification models that predict multiple medical outcomes simultaneously. For example, Maxwell et al [19] and Zhang et al [20] used deep neural networks to predict multiple chronic diseases such as hypertension and diabetes and Lipton et al [21] used recurrent neural networks (RNNs) to classify 128 different diagnoses. Although research has been conducted on developing single-task learning models for ARDS prediction [22-25], thus far no studies have explored multilabel classification models for predicting ARDS. Here, we aimed to perform a deep analysis of how multilabel classification in the clinical setting can take advantage of the underlying dependencies among different diseases to allow for improved performance for the prediction of ARDS in patients over single-label classification models [26]. In addition, although research has been conducted showing that early disease prediction is possible*,* here we also present estimates supporting that early prediction of ARDS is beneficial. Finally, we explore an interesting additional benefit of using neural networks in hospitals and the identification of distinct disease phenotypes.

## Methods

### Data Description

**Figure 1:**
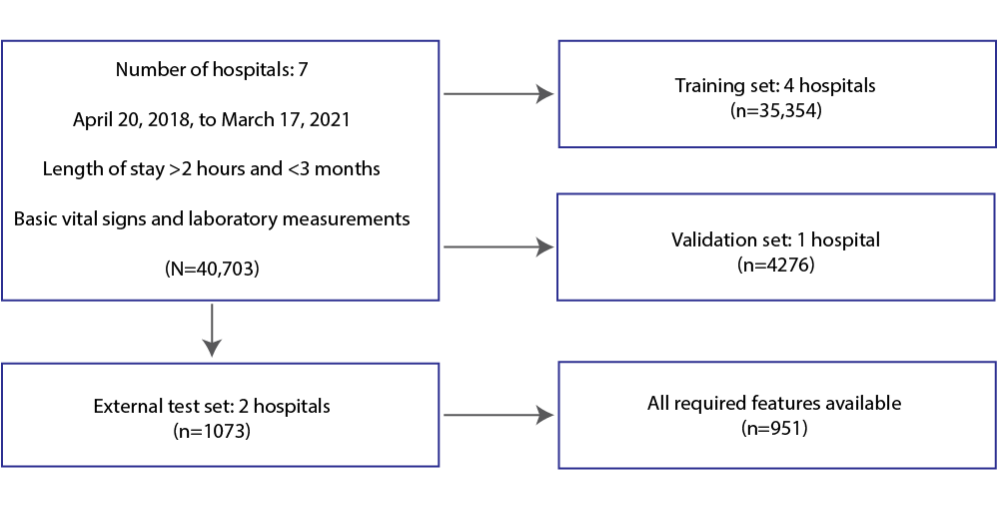
Flowchart of patients. Among 7 hospitals, 40,703 patients met three criteria: (1) admission within the date range (April 20, 2018, to March 17, 2021), (2) length of stay within the range of 2 hours to 3 months, and (3) availability of basic vital signs (blood pressure, heart rate, temperature, respiratory rate, and peripheral oxygen saturation) and laboratory measurements (complete blood count and basic metabolic panel) in the electronic health record. These patients were separated into training, validation, and test sets based on their hospital sites. The test set was limited to those patients with the required features listed in Textbox 1 consisting of age, sex, and basic laboratory measurements, as well as complete blood count with differential.

The data set included 40,703 patient encounters whose care settings included the emergency department, inpatient facility, or ICU. All clinical information was drawn from patient EHR data from 7 different hospitals between April 20, 2018, and March 17, 2021, as shown in Figure 1**.** Data collection was passive, and all patient information was deidentified before the analysis performed in this study. Radiology data were not available. This prevented direct measurement of the Berlin criteria for ARDS [18]. The information collected from each hospital included discharge disposition, demographic data such as age and sex, and time-varying data, including vital signs, laboratory values, oxygen delivery method, medications, and diagnosis times of any conditions present in the health record. These data were extracted as an unordered record of data type, data value, data units, and data collection time, also known as datetime. Preprocessing of these data first involved reordering the data in chronological order under each data type, with 3 equal-length arrays representing the values, units, and datetimes of each measurement. The data were split into training, validation, and external test data sets based on the hospital sites. The training data set used data from 5 hospitals, the validation data set used data from 1 hospital, and the external test data set used data from 2 hospitals. The training and validation data sets were used during the development of the machine learning models, and the external test data set was used to evaluate the models trained on the combination of training and validation data sets.

### Input Features

Model inputs were a defined set of data types, or features, across all hospitals, regardless of the data availability at a particular hospital. Textbox 1 includes all the features used to train the models in this study. The required features are the subset of features, including age, sex, and basic laboratory measurements, as well as complete blood count with differential, used to determine the time the algorithm makes its prediction. Next, these data values were organized into a matrix with features along the first dimension (rows) and discrete time in 20-minute intervals along the second dimension (columns). The first column, column index 0, contains the first time point of any vital sign or laboratory measurement and was considered to be the start of care. The first row was normalized age. The second and third rows were binary indicators for male and female sex. The remaining rows were the time-varying features and their corresponding mask to distinguish missing values from actual zeros (Table 1).

To normalize the features, we carried out a coarse approximation of the mean and SD based on the normal range of these features in the laboratory reports. The center of the normal range was used as the approximate mean value, and half of the difference between the 2 end points of the range was used to approximate the SD. If a feature was missing or not measured, it was set to 0. To let the model distinguish between null values and real values, a new set of features representing the availability mask was vertically appended to the matrix. Each feature row had a corresponding binary mask vector that contained 0s and 1s, representing null values and nonnull values, respectively. During batch training, these matrices were 0 padded on the left side into equal-sized tensors: (batch size, 58 features, 64 timesteps).

**Input features to the machine learning algorithm.**

**Demographics**

- Age (required feature)
- Sex (required feature)

**Vital signs**

- Systolic blood pressure (required feature)
- Diastolic blood pressure (required feature)
- Heart rate (required feature)
- Arterial partial pressure of oxygen
- Respiratory rate (required feature)
- Peripheral oxygen saturation (required feature)
- Temperature (required feature)

**Laboratory results**

- Glucose
- Bilirubin
- White blood cell count (required feature)
- Red blood cell count
- Lymphocytes (required feature)
- Alanine transaminase
- International normalized ratio
- pH
- Blood urea nitrogen
- Creatinine (required feature)
- Platelet
- Neutrophils (required feature)
- Monocytes
- Hematocrit
- Lactate
- Aspartate aminotransferase

**Other measurements**

- Systemic inflammatory response syndrome (the systemic inflammatory response syndrome score is calculated as shown in Table S1 in Multimedia Appendix 1)

**Table 1:** Recurrent neural network features. The first 3 rows are the raw values, the next 3 rows are the corresponding normalized features, and the last 3 rows are the corresponding availability masks to distinguish missing values from actual zeros. The mask is a Boolean vector that is 0 if that measurement is missing and 1 if that measurement is present. It should be noted that this is a subset of the total features and timesteps used for example purposes only. The raw data are also for illustration and not a part of the input feature matrix, which consists of normalized features at 64 timesteps.

			0 minutes		20 minutes		40 minutes		60 minutes
**Raw data**									
	SpO_2_^a^ (%)	90		100		99		80	
	Creatinine (mg/dL)	None		1.8		1.8		1.0	
	WBC^b^ (×10^9^/L)	None		None		None		12	
**Normalized**									
	SpO_2_	–0.5		+0.02		0		–2.3	
	Creatinine	0		+0.2		+0.2		–0.11	
	WBC	0		0		0		+0.15	
**Mask**									
	SpO_2_	1		1		1		1	
	Creatinine	0		1		1		1	
	WBC	0		0		0		1	

^a^SpO_2_: peripheral oxygen saturation.

^b^WBC: white blood cell.

### Model Output and Targets

Additional target labels were chosen for the model that are distinct from ARDS, yet clinically related to it such that a collective representation in the neural network is justified. These labels are shown in Textbox 2 (these output labels will be used throughout the paper hereafter). The model was trained to predict these target labels, also referred to as outcomes, using a binary cross-entropy loss function. The descriptive statistics for the input features for each of the target outcomes are presented in Tables S2 and S3 in Multimedia Appendix 1 for training and test data.

**Clinical outcomes used as target labels for the machine learning algorithm. In total, 13 output labels were mapped to their respective definition.**

**Output label and definition**

- Acute respiratory distress syndrome (ARDS)-1: ARDS defined as having an International Classification of Diseases (ICD) code for ARDS as well as a drop in peripheral oxygen saturation (SpO_2_) below 91%
- ARDS-2: ARDS defined as having an ICD code for ARDS as well as a drop in SpO_2_ below 96%. A direct but broader criterion for ARDS
- ARDS-3: ARDS defined as having an ICD code for ARDS as well as a drop in SpO_2_ below 91% and no mention of a heart failure–related ICD code among prior diagnoses
- ARDS-4: ARDS defined as having an ICD code for ARDS as well as a drop in SpO_2_ below 96% and no mention of a heart failure–related ICD code among prior diagnoses
- ARDS-5: ARDS defined as having an ICD code for ARDS. A direct and simple definition of ARDS
- Sepsis-6: sepsis defined as having an ICD code for sepsis or septic shock as well as a systemic inflammatory response syndrome score >2
- Sepsis-7: sepsis defined as having an ICD code for sepsis or septic shock. A direct and simple definition of sepsis
- Hypoxemia-8: a drop in SpO_2_ below 91% any time during hospitalization
- Hypoxemia-9: a drop in SpO_2_ below 96% any time during hospitalization
- Hypoxemia-10: a drop in SpO_2_ below 91% after algorithm evaluates
- Hypoxemia-11: a drop in SpO_2_ below 96% after algorithm evaluates
- Death-12: in-hospital mortality
- Covid-13: COVID-19 positivity defined as in-hospital COVID-19 positive polymerase chain reaction test or new ICD diagnosis within 7 days before or after admission

### Timing of Algorithm Evaluation

**Figure 2:**
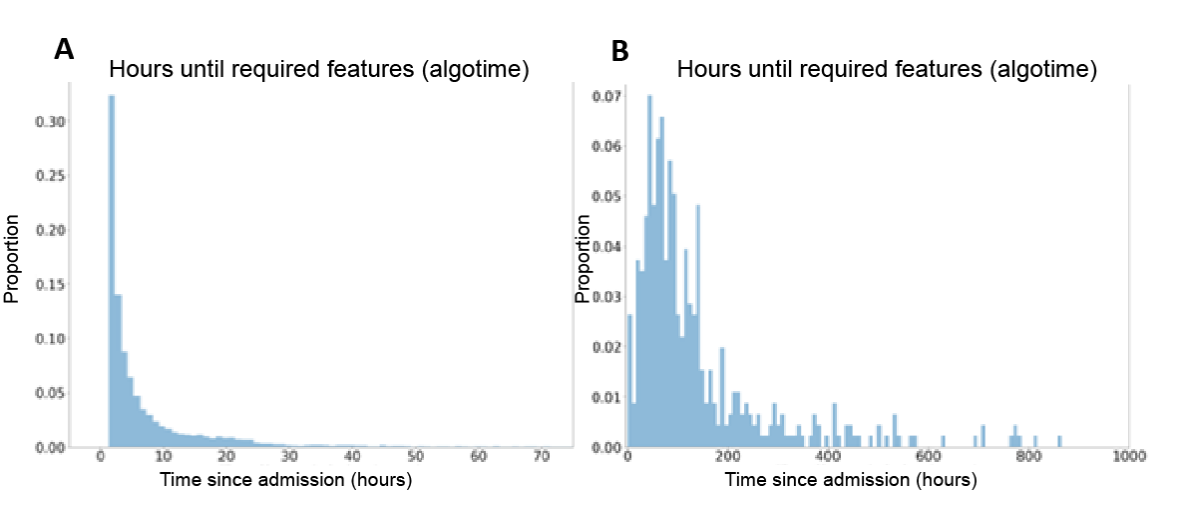
Timing of algorithm and diagnosis. (A) Histogram of number of hours between start of care and the first time point when all required features have been measured at least once (algotime). (B) Histogram of number of hours from start of care until new diagnosis of acute respiratory distress syndrome (ARDS) is entered into the electronic health record. Both histograms are based on the entire data (training+validation+test sets).

For simplicity, we evaluated the algorithm at a single point in time. This time is 2 timesteps (40 minutes) after the first time at which all required features have been measured at least once. At this time, which we refer to as the algotime, the model predicts all the target outcomes of Textbox 2. In the training and validation sets, we used the required features to determine algotime, but in the case of missing features, it defaults to 8 hours after admission. In the test set, we only included patients who had all the required features. With regard to padding, if there are <64 timesteps available before algotime, the input sequence is 0 padded on the left; if there are >64 timesteps available before algotime, only the most recent 64 are taken (no padding). As can be seen in Figure 2**,** on average, the algotime occurred 31 hours after admission and ARDS was clinically diagnosed 139 hours after admission. Thus, the average number of hours between the algotime and the clinical diagnosis of ARDS was 108 hours. It should be noted that the performance statistics reported in this paper correspond to the algotime, not the time of clinical ARDS diagnosis or the end of the hospital stay.

### Benefit Estimation

Thus far, we have defined our machine learning objective as the early prediction of conditions. However, the impact of early predictions on patient health outcomes is more important than the early prediction. To approximate this improvement in the outcome of mortality, we compared mortality rates between patients who received early and late clinical diagnoses of ARDS. We defined early diagnosis and late diagnosis based on when a patient was given a clinical diagnosis of ARDS compared with when the algorithm made a prediction. In other words, a diagnosis for ARDS (using the ARDS-1 definition in Textbox 2 of ARDS International Classification of Diseases [ICD] code and peripheral oxygen saturation [SpO_2_] below 91%) is *early* if it is assigned before the algorithm makes a prediction and *late* if it is assigned after a prediction is made by the algorithm.

### Machine Learning Models

**Figure 3:**
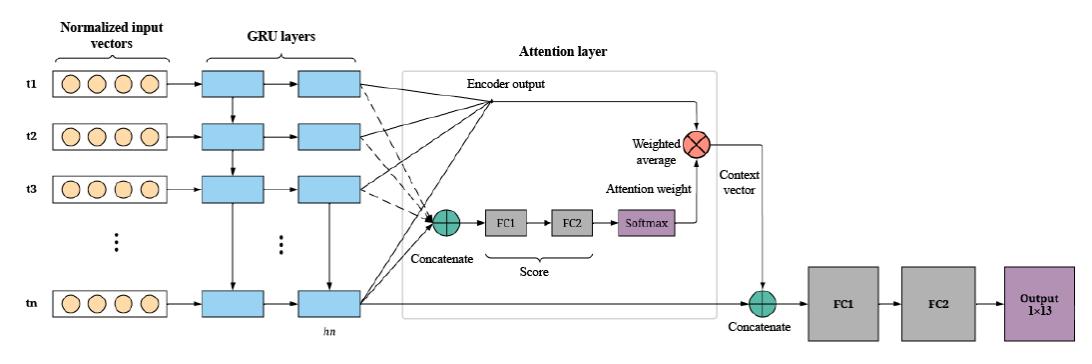
Recurrent neural network model schema. The inputs have been simplified for diagrammatic purposes. Different timesteps of normalized inputs are fed into gated recurrent unit (GRU) layers. The context vector from the attention layer and encoder output from the last GRU are concatenated before feeding them into the first fully connected (FC) layer.

We used an RNN as the main deep learning model for our research. RNNs are a class of artificial neural networks in which connections among nodes form a directed graph along a temporal sequence. RNNs can use their internal memory to process variable length sequences of inputs. The network is capable of learning a mapping function from the inputs over time to an output. It can even learn temporal dependence from the data. All these properties make RNNs a well-suited model for time series data, such as that which is used in this study. The model schema of the RNN used in this research is presented in Figure 3. We used a generic RNN with 4 gated recurrent unit (GRU) layers, an attention module, and 2 fully connected (FC) layers for all numbers of outputs. The RNN was implemented with the PyTorch package (version 1.40) in Python (version 3.6; Python Software Foundation) [27]. For the RNN, the sequence module that was used was a 4-layer GRU [28] with 128 hidden units. Before the sequence of vectors was fed to the GRU, it passed through a normalization layer:


n(v) = *a(v – μ / σ) + b*
** (1)**

Equation 1 is a normalization function that learns the parameters mean *μ*, SD *σ*, scaling *a*, and translation factor *b* used to normalize the sequence of vectors containing the inputs age, sex (Boolean), vital signs, laboratory measurements, and systemic inflammatory response syndrome (SIRS) score before entering the RNN. A soft attention module was used to assign scores to each timestep in the sequence. The scores are intended to be positively correlated with the importance of its respective timestep. A weighted sum of the sequence’s hidden activations was called the context vector. We concatenated the context vector to the final GRU embedding and passed this to a 2-layer feed forward neural network for classification. The intermediate layer before the output logits was a 128D representation of each patient, referred to as the penultimate embedding. Similar to Bahdanau et al [29], the score (equation 2) of the attention neural network was parameterized by a feed forward neural network of the following form:


score(h_l_,h_i_) = K^T^
*tanh*(W_a_ Prelu(W_b_[h_n_,h_i_])) **(2)**

where *tanh* and *Prelu* denote the hyperbolic tangent function and parameterized rectified linear unit nonlinearity functions, respectively; h_n_ denotes the last hidden activation in the GRU; h_i_ denotes each hidden activation in the sequence; i denotes the timestep; [,] denotes concatenation of separate vectors into 1 vector; and K, W_a_, and W_b_ denote learned parameters of the neural network. The whole GRU-RNN, attention module, and classification module were end-to-end differentiable, which enabled optimization from input to output. The attention neural network was a mechanism of the RNN that allowed for higher-quality learning. Instead of summarizing a time series of vectors, the attention neural network assigned each vector a score according to how important the vector was in allowing the model to make a prediction. In this way, the attention network mechanism allowed the RNN to focus on specific parts of the input, thereby enabling improved model performance.

Each point in the RNN model schema represents a neuron. At each layer, the RNN combined the information from the current and previous timesteps to update the activations of the deepest GRU hidden layer. The activation of the last node of the deepest RNN layer is concatenated with the context vector provided by the attention network. The context vector is an importance-weighted average of the deepest layer activations generated by the attention neural network. This concatenated vector is passed through 2 FC layers to generate an output (eg, prediction of ARDS onset). With this RNN schema, the model was trained to predict several target labels simultaneously and to evaluate a loss function based on all targets. We implemented a deep learning method where a single network was trained to output 1 logit per label using a binary cross-entropy loss function [30]. The loss function averages binary cross-entropies against each of the targets in the model, effectively taking into account the output of all 13 tasks. Considering each label a task, this multilabel learning setup can be viewed as a case of multitask learning [31]. Specifically, because all hidden layer parameters are shared among all the targets, it is a hard parameter–sharing variant of multitask learning [32]. Each output logit was independently passed through a sigmoid activation function to produce the final multilabel output [33]. Early stopping was used, based on the ARDS-1 validation performance measured by the area under the receiver operating characteristic curve (AUROC). To explore the relationship between the objective function’s number of targets and final model performance, the lowest 2 AUROC targets were removed successively from each version of the RNN such that the RNN was trained using 13, 11, 9, 7, and 5 targets.

Tree-based models frequently outperform deep learning models in many clinical applications [34]. To ensure that this was not the case in this instance, for comparison, XGBoost (XGB) models for each of the target labels were trained using XGBoost (version 0.81) [35] in Python (version 3.6) [36] and the same feature matrix as the RNN model. The XGB models were trained in a one-versus-all fashion for each target.

### Model Interpretability

Heat maps were produced to visualize attention scores on each time series. Of the 959 patients in the test set, 50 (5.21%) were randomly selected for the following two interpretability analyses: (1) attention scores were visualized across timesteps as heat maps, and (2) the timestep with the highest attention weight generated by the attention network was then further analyzed to visualize each feature’s deviation from the mean in this heavily attended timestep. This method implicitly describes the importance assigned to each feature by the model and provides some insight into model interpretability. The feature vector at that timestep is interpreted as a z-score for the subset of features measured at that particular timestep. For example, a value of 0.5 for the respiratory rate indicates that the respiratory rate is half an SD above the mean.

In addition, Shapley additive explanations (SHAP) force plots for 4 different patients were also generated. The patients represent true positive, true negative, false positive, and false negative cases for ARDS as predicted by the RNN model trained using 13 targets. We used the ARDS-5 definition from Textbox 2 (ARDS defined as having an ICD code for ARDS) for this analysis.

### Clustering

To explore the representations used by the model and to reveal distinct phenotypes among patients with ARDS, we collected the 64D activations produced by the first FC layer as a compressed representation, or embedding, for each patient. To visually display these embeddings, we used principal component analysis. We then used k-means clustering to group each embedding of a patient with ARDS into its unique cluster.

### Statistics

To compare different algorithms and training objectives, we computed the 95% CI around the AUROC using the bootstrapping method [37-39]. These CIs are with respect to the test set (n=959).

### Ethics Approval

All patient data were deidentified in compliance with the Health Insurance Portability and Accountability Act. This study was considered to be of minimal risk for human participants because data collection was passive and did not pose a threat to the participants involved. The project was approved with a waiver of informed consent (20-DASC-122) by an independent institutional review board, Pearl Institutional Review Board.

## Results

### Comparison of RNN Model With XGB Model

The XGB and RNN models were compared across all 13 outputs (Table 2). The performance metric used for comparison was the AUROC. The single RNN model trained to classify 13 outputs outperformed the XGB models trained separately to classify each of the outputs in ARDS and oxygen-related outcomes. The average receiver operating characteristic curves for all targets are also presented in Figure S1 in Multimedia Appendix 1, which further illustrates that the RNN model performs at least as well as the average of all XGB models, with the added advantage that the RNN model benefits from parameter sharing; that is, a single RNN model performs at least as well as the aggregate of 13 XGB models.

**Table 2:** Comparison of performances of the XGB^a^ models and RNN-13^b^ on each of the outcomes. The table provides the AUROC^c^ of each model for each of the labels as well as the sensitivity and specificity. We note that the RNN-13 model outperforms the XGB model on 7 out of 13 outputs.

Labels (prevalence)	XGB				RNN-13		
	AUROC (95% CI)	Sensitivity (95% CI)	Specificity (95% CI)	AUROC (95% CI)		Sensitivity (95% CI)	Specificity (95% CI)
ARDS-1^d,e^ (0.046)	0.797 (0.740-0.851)	0.659 (0.519-0.799)	0.729 (0.7-0.758)	0.842 (0.794-0.888)		0.659 (0.519-0.799)	*0.873 (0.852-0.852)^f^*
ARDS-2 (0.054)	0.700 (0.632-0.768)	0.654 (0.525-0.783)	0.657 (0.626-0.688)	0.791 (0.746-0.836)		0.673 (0.546-0.801)	*0.780 (0.753-0.753)*
ARDS-3 (0.044)	0.786 (0.714-0.856)	0.667 (0.524-0.809)	0.771 (0.744-0.798)	0.845 (0.795-0.894)		0.667 (0.524-0.809)	*0.826 (0.802-0.802)*
ARDS-4 (0.051)	0.748 (0.681-0.81)	0.653 (0.520-0.786)	0.714 (0.685-0.744)	0.812 (0.768-0.858)		0.653 (0.520-0.786)	*0.804 (0.778-0.778)*
ARDS-5 (0.055)	0.701 (0.629-0.77)	0.660 (0.533-0.788)	0.681 (0.651-0.711)	0.795 (0.751-0.839)		0.660 (0.533-0.788)	*0.795 (0.769-0.769)*
Sepsis-6 (0.023)	0.708 (0.604-0.803)	0.682 (0.487-0.876)	0.547 (0.516-0.579)	0.626 (0.533-0.714)		0.682 (0.487-0.876)	0.503 (0.471-0.471)
Sepsis-7 (0.023)	0.707 (0.599-0.798)	0.682 (0.487-0.876)	0.715 (0.686-0.744)	0.586 (0.481-0.681)		0.682 (0.487-0.876)	0.502 (0.469-0.469)
Hypoxemia-8 (0.268)	0.722 (0.684-0.760)	0.658 (0.600-0.716)	0.657 (0.622-0.692)	0.739 (0.708-0.770)		0.651 (0.592-0.709)	0.684 (0.649-0.649)
Hypoxemia-9 (0.799)	0.829 (0.802-0.856)	0.657 (0.623-0.690)	0.876 (0.829-0.922)	0.834 (0.810-0.855)		0.659 (0.625-0.692)	0.839 (0.786-0.786)
Hypoxemia-10 (0.182)	0.643 (0.601-0.688)	0.651 (0.581-0.722)	0.536 (0.501-0.571)	0.638 (0.601-0.673)		0.655 (0.585-0.726)	0.524 (0.489-0.489)
Hypoxemia-11 (0.38)	0.880 (0.861-0.901)	0.654 (0.605-0.703)	0.859 (0.831-0.887)	0.880 (0.862-0.897)		0.651 (0.602-0.700)	0.856 (0.828-0.828)
Death-12 (0.026)	0.761 (0.675-0.841)	0.680 (0.497-0.863)	0.700 (0.671-0.730)	0.700 (0.625-0.768)		0.680 (0.497-0.863)	0.625 (0.594-0.594)
Covid-13 (0.238)	0.805 (0.770-0.840)	0.654 (0.592-0.715)	0.814 (0.786-0.842)	0.673 (0.637-0.714)		0.654 (0.592-0.715)	0.622 (0.587-0.587)

^a^XGB: XGBoost.

^b^RNN-13: the recurrent neural network model that was trained using 13 targets.

^c^AUROC: area under the receiver operating characteristic curve.

^d^ARDS: acute respiratory distress syndrome.

^e^Area under the receiver operating characteristic curve reported in the *Abstract*.

^f^Specificity of the RNN-13 model for the five ARDS labels.

### Benefit of Multitask Learning

**Figure 4:**
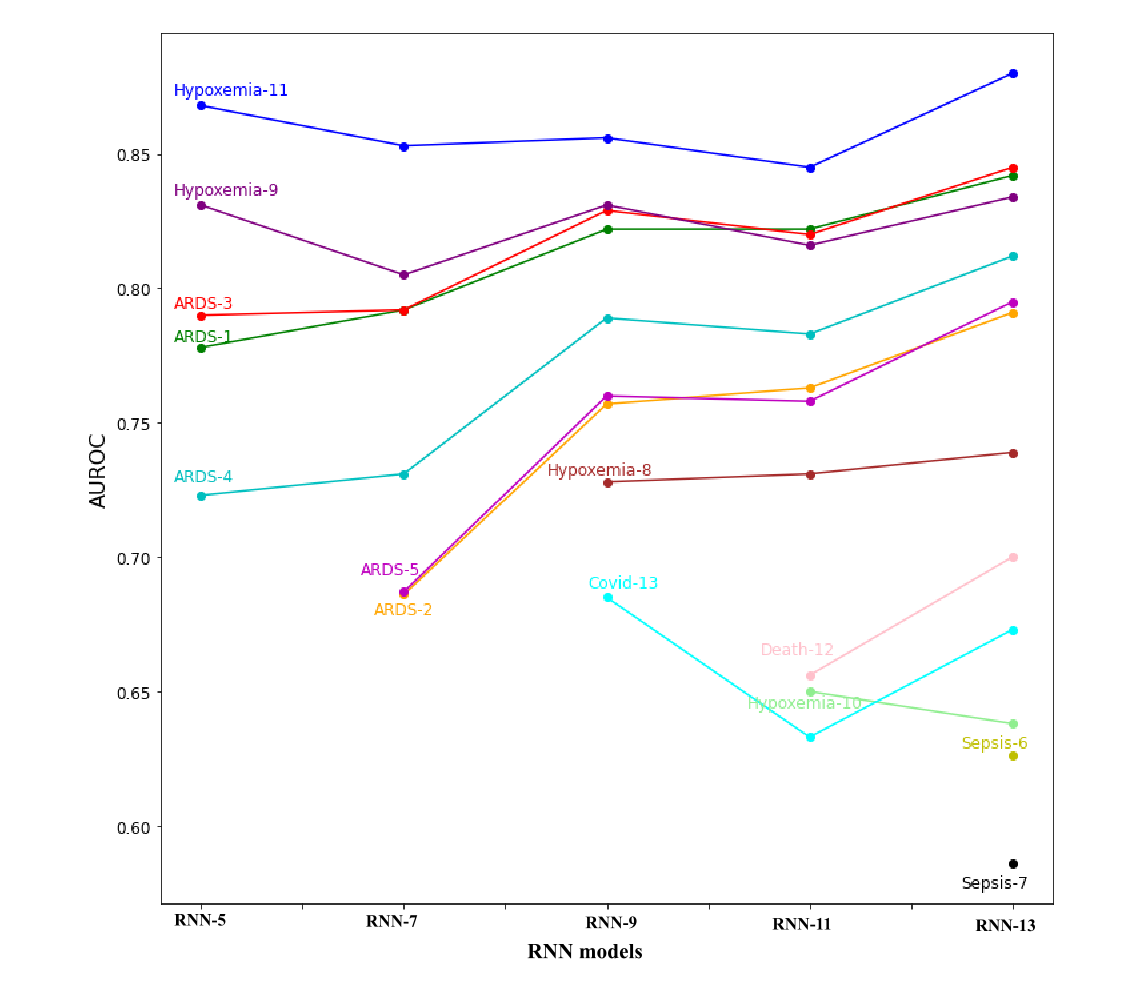
Model performance varies with the number of outcomes predicted during training. External test set area under the receiver operating characteristic curve (AUROC) plotted against the number of targets in the recurrent neural network (RNN) output (eg, RNN-9 refers to an RNN with 9 outputs). From right to left, the worst-performing 2 targets in terms of AUROC are removed to train the next RNN with a smaller number of targets. ARDS: acute respiratory distress syndrome.

An intermediate number of output targets between 1 and 13 were also used to retrain the RNN. Figure 4 shows the maximum AUROC with different subsets of the 13 outcomes used as training targets. For most targets there is a general trend toward overall improvement of the AUROC. This demonstrates that there is some underlying dependency among some of the labels.

### Multitask Learning Converges Training in a Comparable Number of Epochs

**Figure 5:**
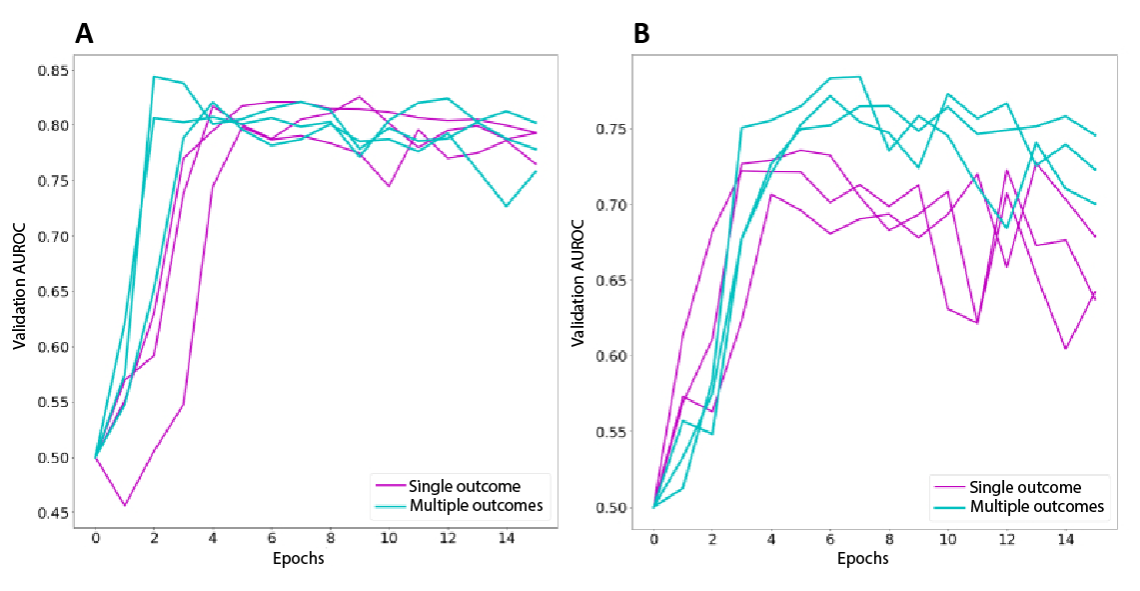
Training on multiple targets converges in similar time to single targets. Learning progress measured using the area under the receiver operating characteristic curve (AUROC) on the validation set. Each line is a different training run with new randomized initial weights and training batches. (A) AUROC predicting ARDS-1 versus number of training epochs. (B) AUROC predicting ARDS-2 versus number of training epochs. ARDS-1: acute respiratory distress syndrome (ARDS) defined as having an International Classification of Diseases code for ARDS as well as a drop in peripheral oxygen saturation below 91%; ARDS-2: ARDS defined as having an International Classification of Diseases code for ARDS as well as a drop in peripheral oxygen saturation below 96%.

The learning quality and efficiency of single- versus multiple-outcome models were evaluated in terms of the rate of improvement of the AUROC on the validation set per each stochastic gradient descent training epoch. We compared the rate of learning between RNNs trained with single targets and RNNs trained with multiple targets to demonstrate that multitask learning does not empirically require longer durations in training than single-learning objectives. The rate of learning was measured as the AUROC of the validation set for each epoch. In Figure 5, the plots of the AUROC of 3 separate randomly initialized training episodes for 15 epochs are shown for ARDS-1 and ARDS-2 (ARDS defined as having an ICD code for ARDS as well as a drop in SpO_2_ below 96%). For these 2 outcomes, the time to reach the maximum validation AUROC in terms of the number of epochs is comparable between single- and multiple-target models.

### ARDS Clustering

**Figure 6:**
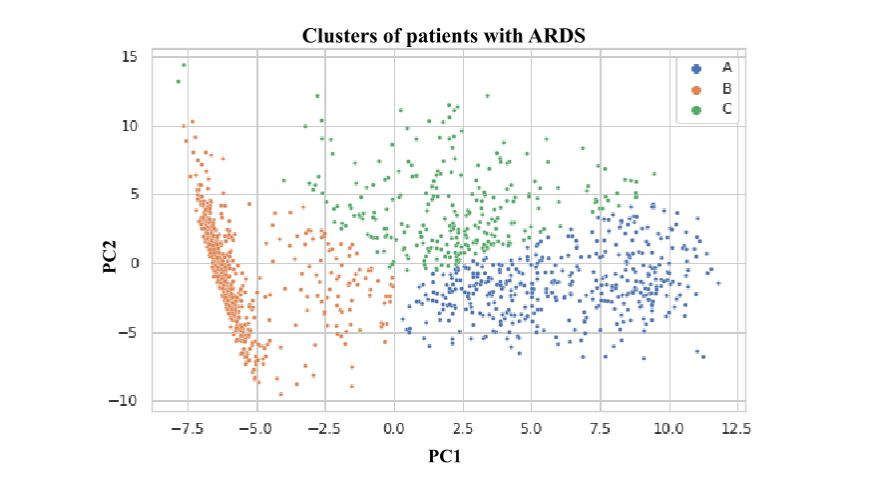
Recurrent neural network representations separate into unique clusters. Clustering the population with acute respiratory distress syndrome (ARDS; n=1278) from the entire data set into 3 different groups A, B, and C by k-means clustering. The dimensions of the embedding vector were reduced using principal component analysis. PC1: principal component 1; PC2: principal component 2.

**Figure 7:**
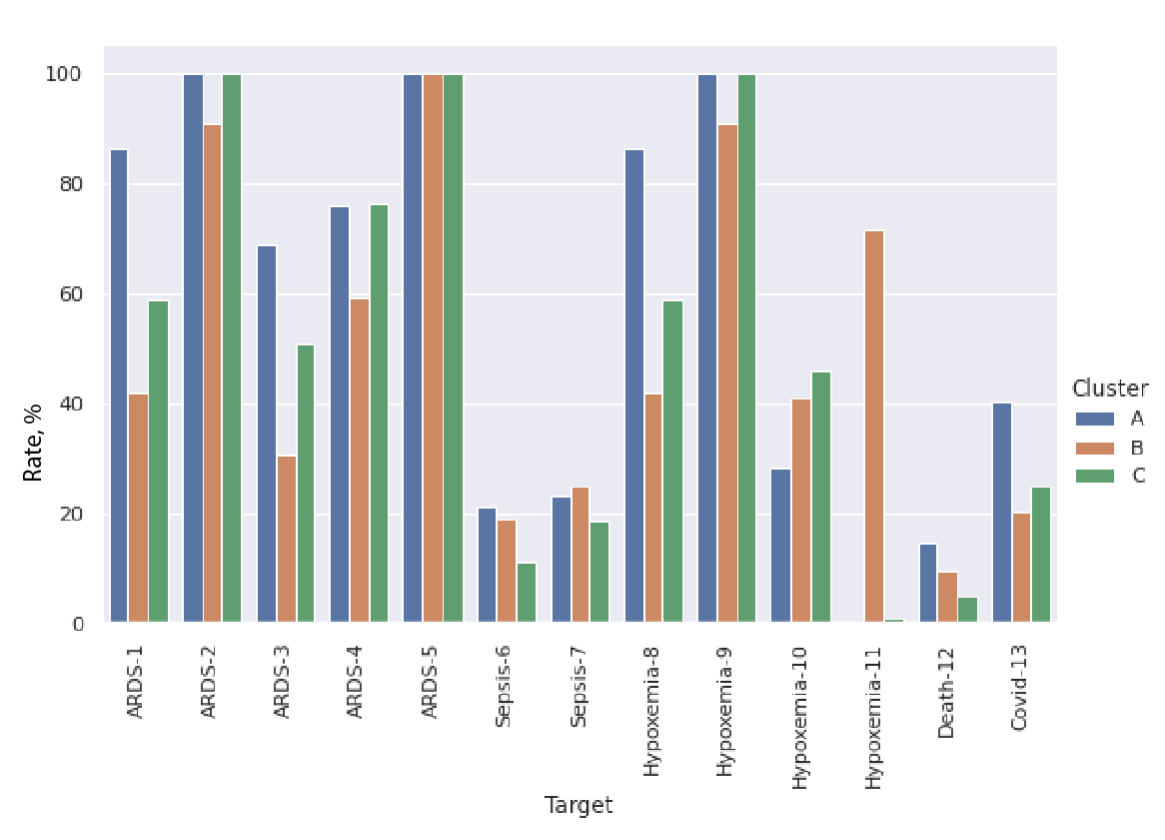
Incidence rates of different targets in each of the clusters. Target 12 is the mortality rate, which is 14.45%, 9.64%, and 4.73% for clusters A, B, and C, respectively. ARDS: acute respiratory distress syndrome.

Figure 6 shows the results of applying the k-means clustering algorithm to find clusters of patients with ARDS on the output of the first FC layer of the RNN model as mentioned in the *Machine Learning Models* section. The k-means algorithm was set to identify 3 clusters because this was the most distinguishable number of clusters in different visualizations of the embeddings. Figure 6 shows a 2D projection of the embeddings using principal component analysis in which the 3 clusters A, B, and C can be seen. Deeper analysis of these clusters is presented in Figure 7**,** which shows a fair amount of variability of the targets among clusters. It can be seen that a drop of SpO_2_ below 91% is more likely to be characterized differently among the clusters than the drop of SpO_2_ below 96% among ARDS targets, which is apparent in higher variability of ARDS-1 and ARDS-3 (ARDS defined as having an ICD code for ARDS as well as a drop in SpO_2_ below 91% and no mention of a heart failure–related ICD code among prior diagnoses) among clusters as opposed to ARDS-2 and ARDS-4 (ARDS defined as having an ICD code for ARDS as well as a drop in SpO_2_ below 96% and no mention of a heart failure–related ICD code among prior diagnoses). The clusters do not seem to be able to distinguish among ARDS symptoms when only the ICD code is used for ARDS prediction (as in ARDS-5). The mortality rate in cluster A is higher than that in the other 2 clusters, which is aligned with the fact that the rates of ARDS-1 and ARDS-3 are also higher in this cluster. Similar relative effects of SpO_2_ <91% versus SpO_2_ <96% can also be seen in hypoxemia targets. Table S4 in Multimedia Appendix 1 shows the distribution of the continuous input features among the 3 clusters. Cluster A shows more noticeable differences with the other 2 clusters when it comes to features such as systolic blood pressure, respiratory rate, neutrophils, lymphocytes, and SIRS, hinting at why the mortality is higher in this group. Note that targets 2, 5, and 9 are the most inclusive (general) of all targets; therefore, it is possible for one or more clusters to be completely enclosed by these targets, resulting in a rate of 100% (Figure 7). These inclusive targets are included to improve parameter sharing in the model for different outcomes.

### Benefit Estimation

From our benefit estimation case study, we found that the mortality rate for patients who were diagnosed early with ARDS was 5.3% (14/266), whereas for those diagnosed late with ARDS, the mortality rate was 11.7% (116/995). The Fisher exact test statistic value was 0.002 for the 6.3% mortality benefit of using the algorithm’s prediction for the early diagnosis group. For reference, the baseline mortality rate of patients without ARDS was 1.66% (656/39,442) and that of patients with ARDS was 10.31% (130/1261).

### Model Interpretability

**Figure 8:**
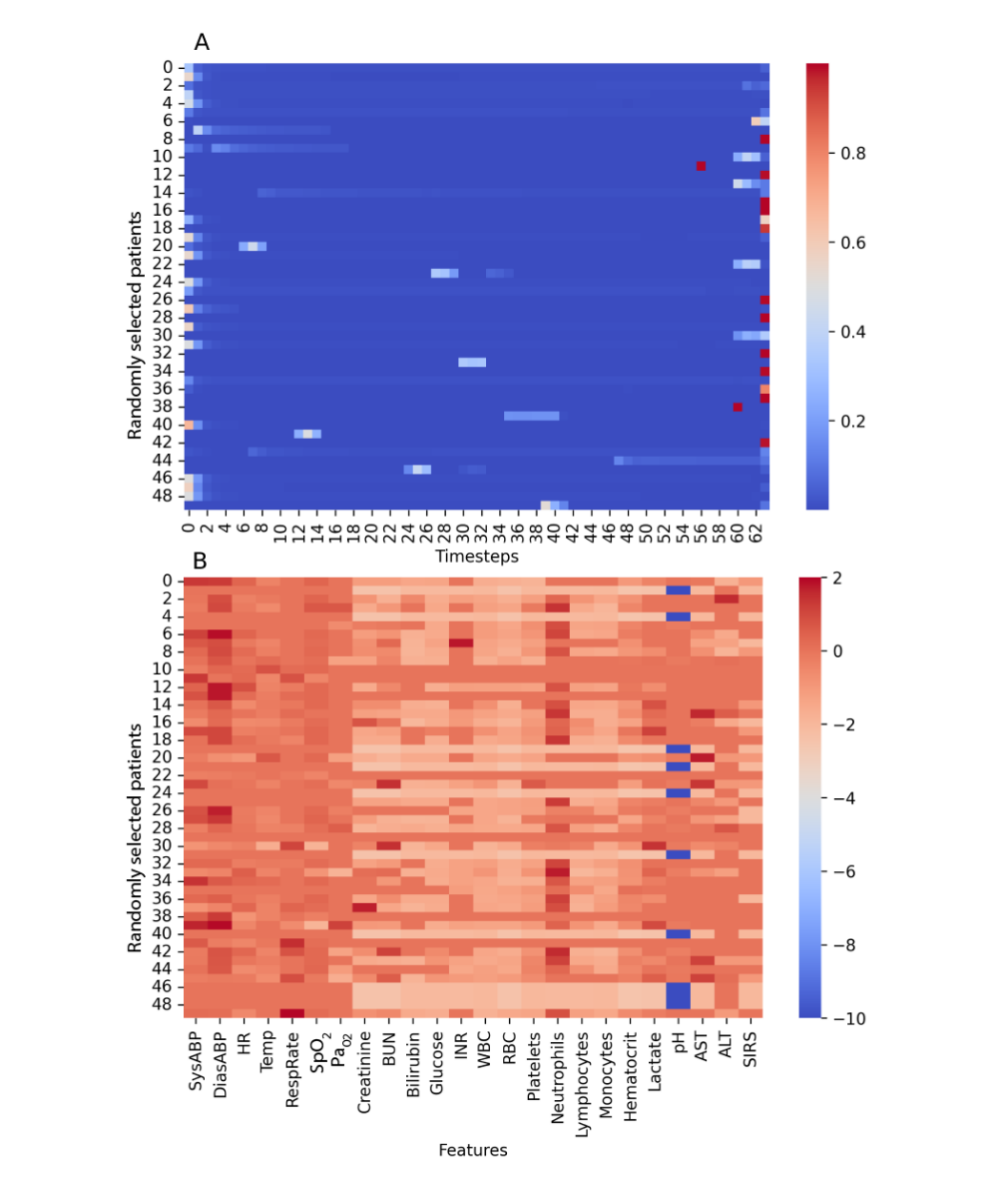
Attention heat maps. Each row along the y-axis is a patient. (A) Attention weights for timesteps. The heat map visualizes the attention weights on 50 randomly selected patients for the 64-timestep inputs to the recurrent neural network model. (B) Attention scores for features. The heat map visualizes the calculated z-score for every time-varying feature at the timestep with the greatest attention weight for the same set of patients as in part A. Red denotes a higher value or deviation in the positive direction; blue denotes a lower value or deviation in the negative direction. ALT: alanine transaminase; AST: aspartate aminotransferase; BUN: blood urea nitrogen; DiasABP: diastolic ambulatory blood pressure; HR: heart rate; INR: international normalized ratio; Pa_O2_: arterial partial pressure of oxygen; RBC: red blood cell count; RespRate: respiratory rate; SIRS: systemic inflammatory response syndrome; SpO_2_: peripheral oxygen saturation; SysABP: systolic ambulatory blood pressure; Temp: temperature; WBC: white blood cell count.

A visualization of the attention weights for different timesteps of the input sequence is shown in Figure 8A in which we observe variability in the distribution of the attention weights within the 64-timestep window. For some of the samples, the attention weights are higher toward the beginning of the sequence, whereas for others they are higher toward the end of the sequence. There are also cases where the attention weight is moderately higher in the middle of the sequence. The cases for which the attention weight is higher toward the end of the sequence represent the situation in which the most recent measurements with respect to the event of interest are more important. The cases for which the attention weights are higher toward the beginning of the sequence represent the situation in which the most relevant temporal data are near the beginning of, or before, the 64-timestep window. In this scenario, it is probably the attention network that is amplifying the signal from those early timesteps because without the attention network, GRUs alone will have a gradual decay of older timesteps. In the samples in which the attention weights are higher in the middle, there likely exists an intermediate timestep that has abnormal values that is emphasized more by the network. Figure 8B shows the calculated attention scores for specific features for the same patients as accounted for in Figure 8A. These scores were obtained by calculating the feature’s z-score at the timestep with the highest attention weight. The figure is shown for all time-varying features. The figure reveals how every feature affects the model output. For example, high values of respiratory rate and low values of pH had a positive impact on the model.

From the SHAP force plots in Figure S2 in Multimedia Appendix 1, one can see the most influential features for a given patient. Red denotes the positive direction of influence on the ARDS-5 output, whereas blue denotes the negative direction of influence on the ARDS-5 output. The length of the arrow denotes the magnitude of SHAP values. The value in bold is the actual model output, which is then transformed into probability space to give the final output between 0 and 1. SpO_2_ is an important feature, both to increase the probability of ARDS when SpO_2_ is low (A) and to lower the probability of ARDS when SpO_2_ is high (B). Low SpO_2_ combined with high respiratory rate is the likely contributor to a false positive (C) in the context of a patient with poorly controlled diabetes (ie, blood glucose level=505 mg/dL and likely tachypnea of diabetic ketoacidosis) [40,41]. A normal SIRS score and normal neutrophil percentage in the absence of strongly positive features results in a false negative (D).

## Discussion

### Principal Findings

In this study, we described the development of a deep learning model for predicting multiple outcomes by simultaneously using the same set of input features. We showed that the RNN model trained to predict 13 outcomes simultaneously generalized better on ARDS outcomes than XGB models trained to predict individual outcomes. We showed that this improvement was proportional to the number of targets predicted by the RNN. This reinforces our conclusion that training the RNN model on a larger set of outcomes improves generalization. We hypothesize that multitask learning generalizes better in part because of parameter sharing, which has a regularizing effect, and information sharing across outcomes, which learns richer representations [42]. We would also like to emphasize that the intention of this paper was not to advance state-of-the-art multitask learning but to provide evidence that multitask learning is beneficial for early prediction of ARDS using only EHR data.

We used an RNN in this study because of its ability to use its internal memory to process variable length sequences of inputs, learn temporal dependence from the data, and share representations for an arbitrary number of outputs. We used a generic RNN with 4 GRU layers, an attention module, and 2 FC layers for all numbers of outputs. We experimented with various RNN architectures, varying the parameters such as the number of layers and hidden units. From our light grid search, we found that the RNN model architecture used in this paper performed best for our use case. In addition, the attention module seems to be an important part of the architecture in making the prediction because without it, the performance of the RNN dropped significantly for multiple targets as seen in Table S5 in Multimedia Appendix 1.

To compare the RNN with other algorithms, we used XGB because of its ability to handle missing or null values and its current dominance in industrial applications. As EHR data often have a high level of missing values because of variability in data acquisition and recording habits in the live clinical environment, this attribute of XGB is appealing. We trained multiple XGB models separately on the same input to classify different outcomes independently. We performed a grid search for hyperparameter optimization, tuning parameters such as tree depth and learning rate. Table S6 in Multimedia Appendix 1 shows the hyperparameters that were used for the grid search for the RNN and XGB models.

We also demonstrated an application of cluster analysis to probe deep learning models for clinical insights. Our analysis of the total population with ARDS uncovered 3 distinct populations, 2 of which have similarly high mortality rates but different clinical presentations. Recent studies have corroborated similar results in populations with COVID-19 in which 2 distinct phenotypes of ARDS were found with similar respiratory dynamics but 2-fold difference in odds of 28-day mortality [43]. With the methods outlined in this study, phenotype discovery would be an additional benefit that can be automatically applied to an arbitrarily large number of outcomes predicted.

To connect our machine learning findings with real-world clinical effects, we compared the mortality rates between patients diagnosed earlier with ARDS and patients diagnosed later with ARDS relative to the algorithm’s prediction time. Our estimation showed that the mortality rate in the population diagnosed early with ARDS was almost half of that in the population diagnosed late with ARDS. Finally, to make our model more interpretable, we provide 2 heat maps attempting to visualize the attention score on each time series as well as 4 SHAP force plots presenting our case analyses regarding success and failure prediction.

Although we—and other researchers—have previously developed single-task machine learning models for predicting ARDS in different cohorts of hospitalized patients, to our knowledge, this is the first study to develop a multitask deep learning model for ARDS prediction [22-25]. Although previous studies have reported the development of high-performing ARDS prediction models, we intentionally do not make direct comparisons of model performance between our model and previous models for several reasons. The first is that to demonstrate that multitask learning improves performance over single-task learning, the models should ideally be trained and tested in a similar manner and on the same data sets. Comparisons with other published models may not provide any useful information on the direct benefit of using multitask learning models for ARDS prediction. Another point of consideration is that we used several subtypes of ARDS in our study; therefore, direct comparison against metrics from other studies that may use different ARDS definitions may not be fruitful.

Real-world clinical utility of such machine learning algorithms would need to be demonstrated through a multicenter prospective clinical study. We have previously developed and demonstrated the real-world impact of a sepsis prediction algorithm (InSight) on patient outcomes in a multicenter clinical validation study [44]. Although we performed retrospective validation on an external test set and demonstrated good performance of our algorithm in this study, ideally, the algorithm should be tested at multiple hospitals that vary by geographic location and patient demographic characteristics. Demonstrating a reduction in length of stay and improved outcomes of patients with ARDS through a clinical study would pave the way for deployment of the algorithm at medical institutions.

This study includes several limitations. In many hospital systems, radiology images and radiology reports are kept in a software system separate from the EHR. Ideally, we would prefer to confirm ARDS ICD codes by verifying the presence of bilateral lung infiltrates on chest imaging. Our inputs only included demographics, vital signs, and laboratory information. Future work should therefore incorporate EHR as well as imaging data. Our data set spans the emergency department, inpatient, and ICU settings and prescribes a single early time point for prediction. This could be a factor in the low AUROC for sepsis predictions, which prior studies have shown to be reliably accurate in the ICU setting [12,44]. This discrepancy warrants further investigation. In addition, we did not have reliable data on race and ethnicity of the patient population. Future studies would also benefit from training the models to predict the additional output of respiratory support intervention beyond the level of a nonrebreather mask [45]. Finally, because this is a retrospective study, we are not able to determine the performance of our algorithm in a prospective clinical setting. Prospective testing is essential to determine how clinicians will respond to predictions of various outcomes. It is also important to determine whether our predictions can affect patient outcomes or resource allocation. Our work here is meant to serve as a reference for future research directions in establishing the most beneficial role for machine learning algorithms in the health care ecosystem and expanding the capabilities of machine learning in health care. Future research could also incorporate examining more state-of-the-art RNN architectures such as transformers that may have better performance for long sequence data processing.

### Conclusions

We present a novel multitask deep learning model for predicting ARDS in hospitalized patients. Our results demonstrate that, based on the same input features, the higher the number of related outcomes predicted by our model, the better the performance on most outcomes. We demonstrate the clinical utility of our model by calculating the sensitivity, specificity, and AUROC of various iterations of the model on 2 external test sets and explore the interpretability of our model by visualizing attention weights using heat maps and SHAP for global and local model interpretability. Early prediction of ARDS, together with the stratification of patients into different subgroups based on different clinical presentations, will enable clinicians to take appropriate action to prevent the deterioration of a patient’s condition, which should in turn improve patient outcomes and mortality or morbidity rates of ARDS.

## Appendix

Multimedia Appendix 1 Supplementary tables and figures.

## Abbreviations

ARDS: acute respiratory distress syndrome
AUROC: area under the receiver operating characteristic curve
EHR: electronic health record
FC: fully connected
GRU: gated recurrent unit
ICD: International Classification of Diseases
ICU: intensive care unit
RNN: recurrent neural network
SHAP: Shapley additive explanations
SIRS: systemic inflammatory response syndrome
SpO_2_: peripheral oxygen saturation
XGB: XGBoost

## References

[1] Matthay MA, Zemans RL, Zimmerman GA, Arabi YM, Beitler JR, Mercat A, Herridge M, Randolph AG, Calfee CS (2019). Acute respiratory distress syndrome. Nat Rev Dis Primers.

[2] Sweeney RM, McAuley DF (2016). Acute respiratory distress syndrome. Lancet.

[3] Ferguson ND, Fan E, Camporota L, Antonelli M, Anzueto A, Beale R, Brochard L, Brower R, Esteban A, Gattinoni L, Rhodes A, Slutsky AS, Vincent J, Rubenfeld GD, Thompson BT, Ranieri VM (2012). The Berlin definition of ARDS: an expanded rationale, justification, and supplementary material. Intensive Care Med.

[4] Riviello ED, Kiviri W, Twagirumugabe T, Mueller A, Banner-Goodspeed VM, Officer L, Novack V, Mutumwinka M, Talmor DS, Fowler RA (2016). Hospital incidence and outcomes of the acute respiratory distress syndrome using the kigali modification of the Berlin definition. Am J Respir Crit Care Med.

[5] Gajic O, Dabbagh O, Park PK, Adesanya A, Chang SY, Hou P, Anderson H, Hoth JJ, Mikkelsen ME, Gentile NT, Gong MN, Talmor D, Bajwa E, Watkins TR, Festic E, Yilmaz M, Iscimen R, Kaufman DA, Esper AM, Sadikot R, Douglas I, Sevransky J (2011). Early identification of patients at risk of acute lung injury. Am J Respir Crit Care Med.

[6] Soto GJ, Kor DJ, Park PK, Hou PC, Kaufman DA, Kim M, Yadav H, Teman N, Hsu MC, Shvilkina T, Grewal Y, De Aguirre M, Gunda S, Gajic O, Gong MN (2016). Lung injury prediction score in hospitalized patients at risk of acute respiratory distress syndrome. Crit Care Med.

[7] Levitt J, Calfee C, Goldstein BA, Vojnik R, Matthay MA (2013). Early acute lung injury. Crit Care Med.

[8] Seitz K, Caldwell E, Hough CL (2020). Fluid management in ARDS: an evaluation of current practice and the association between early diuretic use and hospital mortality. J Intensive Care.

[9] Malhotra A (2007). Low-tidal-volume ventilation in the acute respiratory distress syndrome. N Engl J Med.

[10] Esteva A, Robicquet A, Ramsundar B, Kuleshov V, DePristo M, Chou K, Cui C, Corrado G, Thrun S, Dean J (2019). A guide to deep learning in healthcare. Nat Med.

[11] Radhachandran A, Garikipati A, Zelin NS, Pellegrini E, Ghandian S, Calvert J, Hoffman J, Mao Q, Das R (2021). Prediction of short-term mortality in acute heart failure patients using minimal electronic health record data. BioData Min.

[12] Mao Q, Jay M, Hoffman JL, Calvert J, Barton C, Shimabukuro D, Shieh L, Chettipally U, Fletcher G, Kerem Y, Zhou Y, Das R (2018). Multicentre validation of a sepsis prediction algorithm using only vital sign data in the emergency department, general ward and ICU. BMJ Open.

[13] Mohamadlou H, Lynn-Palevsky A, Barton C, Chettipally U, Shieh L, Calvert J, Saber NR, Das R (2018). Prediction of acute kidney injury with a machine learning algorithm using electronic health record data. Can J Kidney Health Dis.

[14] Ryan L, Mataraso S, Siefkas A, Pellegrini E, Barnes G, Green-Saxena A, Hoffman J, Calvert J, Das R (2021). A machine learning approach to predict deep venous thrombosis among hospitalized patients. Clin Appl Thromb Hemost.

[15] Giang C, Calvert J, Rahmani K, Barnes G, Siefkas A, Green-Saxena A, Hoffman J, Mao Q, Das R (2021). Predicting ventilator-associated pneumonia with machine learning. Medicine (Baltimore).

[16] Rahmani K, Garikipati A, Barnes G, Hoffman J, Calvert J, Mao Q, Das R (2022). Early prediction of central line associated bloodstream infection using machine learning. Am J Infect Control.

[17] Coudroy R, Frat J, Boissier F, Contou D, Robert R, Thille AW (2018). Early identification of acute respiratory distress syndrome in the absence of positive pressure ventilation. Crit Care Med.

[18] Ranieri VM, Rubenfeld GD, Thompson BT, Ferguson ND, Caldwell E, Fan E, Camporota L, Slutsky AS, ARDS Definition Task Force (2012). Acute respiratory distress syndrome: the Berlin definition. JAMA.

[19] Maxwell A, Li R, Yang B, Weng H, Ou A, Hong H, Zhou Z, Gong P, Zhang C (2017). Deep learning architectures for multi-label classification of intelligent health risk prediction. BMC Bioinformatics.

[20] Zhang X, Zhao H, Zhang S, Li R (2019). A novel deep neural network model for multi-label chronic disease prediction. Front Genet.

[21] Lipton Z, Kale D, Elkan C, Wetzel R (2016). Learning to diagnose with LSTM recurrent neural networks. Proceedings of the 4th International Conference on Learning Representations, ICLR 2016.

[22] Zeiberg D, Prahlad T, Nallamothu BK, Iwashyna TJ, Wiens J, Sjoding MW (2019). Machine learning for patient risk stratification for acute respiratory distress syndrome. PLoS One.

[23] Singhal L, Garg Y, Yang P, Tabaie A, Wong AI, Mohammed A, Chinthala L, Kadaria D, Sodhi A, Holder AL, Esper A, Blum JM, Davis RL, Clifford GD, Martin GS, Kamaleswaran R (2021). eARDS: a multi-center validation of an interpretable machine learning algorithm of early onset Acute Respiratory Distress Syndrome (ARDS) among critically ill adults with COVID-19. PLoS One.

[24] Lam C, Tso C, Green-Saxena A, Pellegrini E, Iqbal Z, Evans D, Hoffman J, Calvert J, Mao Q, Das R (2021). Semisupervised deep learning techniques for predicting acute respiratory distress syndrome from time-series clinical data: model development and validation study. JMIR Form Res.

[25] Le S, Pellegrini E, Green-Saxena A, Summers C, Hoffman J, Calvert J, Das R (2020). Supervised machine learning for the early prediction of acute respiratory distress syndrome (ARDS). J Crit Care.

[26] Read J, Perez-Cruz F Deep learning for multi-label classification. arXiv..

[27] Paszke A, Gross S, Chintala S, Chanan G, Yang E, DeVito Z, Lin Z, Desmaison A, Antiga L, Lerer A (2017). Automatic differentiation in PyTorch. Proceedings of the 31st Conference on Neural Information Processing Systems (NIPS 2017).

[28] Chung J, Gulcehre C, Cho K, Bengio Y (2014). Empirical evaluation of gated recurrent neural networks on sequence modeling. Proceedings of the NIPS 2014 Workshop on Deep Learning.

[29] Bahdanau D, Cho K, Bengio Y Neural machine translation by jointly learning to align and translate. arXiv..

[30] Good I (1956). Some terminology and notation in information theory. Proc IEE C Monogr UK.

[31] Zhang Y, Yang Q (2021). A survey on multi-task learning. IEEE Trans Knowl Data Eng.

[32] Ruder S (2017). An overview of multi-task learning in deep neural networks. arXiv.

[33] Ridnik T, Ben-Baruch E, Zamir N, Noy A, Friedman I, Protter M, Zelnik-Manor L (2021). Asymmetric loss for multi-label classification. Proceedings of the 2021 IEEE/CVF International Conference on Computer Vision (ICCV).

[34] Lundberg SM, Erion G, Chen H, DeGrave A, Prutkin JM, Nair B, Katz R, Himmelfarb J, Bansal N, Lee S (2020). From local explanations to global understanding with explainable AI for trees. Nat Mach Intell.

[35] Chen T, Guestrin C (2016). XGBoost: a scalable tree boosting system. Proceedings of the 22nd ACM SIGKDD International Conference on Knowledge Discovery and Data Mining.

[36] XGBoost Python Package. dmlc XGBoost.

[37] Lundberg S, Lee S-I (2017). A unified approach to interpreting model predictions. Proceedings of the 31st International Conference on Neural Information Processing Systems.

[38] Bertail P, Clémençcon S, Vayatis N (2008). On Bootstrapping the ROC curve. Proceedings of the 21st International Conference on Neural Information Processing Systems.

[39] Liu H, Li G, Cumberland W, Wu T (2005). Testing statistical significance of the area under a receiving operating characteristics curve for repeated measures design with bootstrapping. J Data Sci.

[40] Gallo de Moraes A, Surani S (2019). Effects of diabetic ketoacidosis in the respiratory system. World J Diabetes.

[41] Westerberg DP (2013). Diabetic ketoacidosis: evaluation and treatment. Am Fam Physician.

[42] Nam J, Loza Mencía E, Kim H, Fürnkranz J (2017). Maximizing subset accuracy with recurrent neural networks in multi-label classification. Proceedings of theAdvances in Neural Information Processing Systems 30: Annual Conference on Neural Information Processing Systems 2017.

[43] Ranjeva S, Pinciroli R, Hodell E, Mueller A, Hardin CC, Thompson BT, Berra L (2021). Identifying clinical and biochemical phenotypes in acute respiratory distress syndrome secondary to coronavirus disease-2019. EClinicalMedicine.

[44] Desautels T, Calvert J, Hoffman J, Jay M, Kerem Y, Shieh L, Shimabukuro D, Chettipally U, Feldman MD, Barton C, Wales DJ, Das R (2016). Prediction of sepsis in the intensive care unit with minimal electronic health record data: a machine learning approach. JMIR Med Inform.

[45] Wong A, Cheung P, Kamaleswaran R, Martin GS, Holder AL (2020). Machine learning methods to predict acute respiratory failure and acute respiratory distress syndrome. Front Big Data.

