# Supplementary material for: Multitask Learning With Recurrent Neural Networks for Acute Respiratory Distress Syndrome Prediction Using Only Electronic Health Record Data: Model Development and Validation Study
Source: JMIR Med Inform. 2022 Jun 15;10(6):e36202. doi: 10.2196/36202 (PMC9244659; doi:10.2196/36202)
Supplement: Multimedia Appendix 1 [file medinform_v10i6e36202_app1.docx]

**Multimedia Appendix 1: Supplementary Materials**

**Table S1.** Calculation of the SIRS score. The score is the sum of each of the individual scores shown in this table: SIRS = HR + TEMP + RR + WBC. SIRS Criteria (total score >=2 meets SIRS definition)

|  | Condition | Score |
| --- | --- | --- |
| HR | Heart Rate <= 90 (BPM) | 0 |
|  | Heart Rate > 90 (BPM) | 1 |
| TEMP | 35 (ºC) < Temperature <= 38 (ºC) | 0 |
|  | Temperature <= 35 (ºC) or Temperature > 38 (ºC) | 1 |
| RR | Respiratory Rate <= 20 (BPM) | 0 |
|  | Respiratory Rate > 20 (BPM) | 1 |
| WBC | 4 (10^3^/μL) <= White Blood Cell Count <= 12 (10^3^/μL) | 0 |
|  | White Blood Cell Count < 4 (10^3^/μL) or White Blood Cell Count >12 (10^3^/μL) | 1 |

**Table S2.** Descriptive statistics for training dataset. The values were obtained using the overall average and standard deviation of all times series values for all patients. The values for male and female variables are counts. The ALL column is the overall feature mean (std) or count for training data. Note that the sets of patients in each target are not necessarily mutually exclusive.

|  | ARDS_1 | ARDS_2 | ARDS  _3 | ARDS_4 | ARDS_5 | Sepsis_6 | Sepsis_7 | Hypoxemia_8 | Hypoxemia_9 | Hypoxemia_10 | Hypoxemia_11 | Death | Covid19 | ALL |
| --- | --- | --- | --- | --- | --- | --- | --- | --- | --- | --- | --- | --- | --- | --- |
| Age (years) | 64.25 (15.02) | 64.5 (15.01) | 63.17 (15.13) | 63.17 (15.48) | 64.34 (15.03) | 61.18 (17.43) | 62.39 (17.25) | 62.58 (17.69) | 60.54 (18.79) | 63.9 (17.15) | 60.54 (19.2) | 72.1 (15.3) | 63.89 (17.61) | 55.25 (20.95) |
| SystolicBP (mmHg) | 126.5 (20.84) | 128.72 (22.07) | 125.89 (19.88) | 127.32 (20.54) | 129.16 (22.36) | 124.57 (21.87) | 125.58 (22.26) | 128.05 (20.61) | 131.04 (20.9) | 128.87 (21.51) | 133.12 (22.49) | 125.87 (22.83) | 130.15 (20.02) | 131.18 (20.6) |
| DiastolicBP (mmHg) | 71.86 (11.64) | 72.77 (11.91) | 71.83 (11.12) | 72.61 (11.48) | 72.95 (11.84) | 71.47 (12.1) | 71.83 (12.11) | 72.41 (11.06) | 74.18 (10.88) | 72.61 (11.48) | 75.02 (11.93) | 70.13 (11.91) | 73.32 (10.89) | 75.08 (10.78) |
| HR (BPM) | 90.08 (18.87) | 88.9 (19.0) | 91.22 (18.96) | 90.35 (19.3) | 88.55 (18.9) | 95.69 (18.7) | 91.74 (19.29) | 87.9 (17.26) | 85.95 (16.87) | 87.3 (17.43) | 85.77 (17.95) | 90.81 (20.2) | 87.67 (17.17) | 86.13 (16.82) |
| Temp (ºC) | 36.94 (0.63) | 36.9 (0.58) | 36.97 (0.64) | 36.94 (0.6) | 36.89 (0.58) | 37.15 (0.81) | 37.05 (0.75) | 36.84 (0.56) | 36.84 (0.48) | 36.85 (0.58) | 36.84 (0.51) | 36.81 (0.81) | 37.13 (0.69) | 36.84 (0.45) |
| RespRate (BPM) | 20.18 (3.81) | 19.83 (3.62) | 20.09 (3.85) | 19.84 (3.75) | 19.77 (3.6) | 19.43 (3.44) | 19.05 (3.2) | 19.22 (3.45) | 18.44 (2.73) | 18.87 (3.08) | 18.32 (2.45) | 20.35 (4.15) | 19.93 (3.53) | 18.22 (2.57) |
| SpO2 (%) | 95.22 (3.05) | 95.81 (2.79) | 95.2 (2.96) | 95.72 (2.78) | 95.93 (2.8) | 96.3 (2.47) | 96.41 (2.45) | 95.58 (2.98) | 96.45 (2.22) | 96.51 (2.0) | 98.07 (1.19) | 95.68 (3.64) | 95.3 (3.17) | 97.06 (2.13) |
| PaO2 (mmHg) | 84.97 (22.15) | 85.28 (22.62) | 85.1 (22.34) | 84.99 (23.19) | 85.22 (22.54) | 93.08 (25.2) | 92.65 (25.23) | 90.5 (23.57) | 91.89 (23.61) | 94.83 (24.23) | 103.4 (24.48) | 89.62 (22.92) | 80.09 (23.47) | 92.83 (23.93) |
| Creatinine (mg/dL) | 1.16 (0.57) | 1.13 (0.54) | 1.11 (0.55) | 1.08 (0.52) | 1.13 (0.53) | 1.11 (0.54) | 1.1 (0.53) | 1.11 (0.52) | 1.05 (0.47) | 1.11 (0.51) | 1.03 (0.47) | 1.22 (0.57) | 1.1 (0.5) | 1.01 (0.44) |
| BUN (mg/dL) | 22.48 (10.98) | 22.89 (11.07) | 21.71 (10.82) | 21.45 (10.59) | 22.92 (11.12) | 20.96 (10.62) | 21.06 (10.54) | 20.45 (10.61) | 18.85 (9.92) | 20.61 (10.47) | 19.03 (10.03) | 24.2 (10.94) | 20.45 (10.59) | 17.5 (9.48) |
| Bilirubin (mg/dL) | 0.77 (0.59) | 0.75 (0.56) | 0.74 (0.57) | 0.71 (0.54) | 0.75 (0.56) | 0.81 (0.65) | 0.79 (0.61) | 0.72 (0.58) | 0.68 (0.55) | 0.73 (0.57) | 0.69 (0.56) | 0.89 (0.72) | 0.69 (0.47) | 0.64 (0.52) |
| Glucose (mg/dL) | 150.56 (70.39) | 151.58 (78.11) | 149.44 (72.28) | 148.9 (75.69) | 152.81 (85.93) | 153.53 (81.15) | 150.32 (80.21) | 146.39 (78.01) | 140.49 (75.55) | 147.01 (80.34) | 138.74 (78.02) | 154.94 (74.91) | 146.11 (73.54) | 134.82 (75.14) |
| INR ^1^ | 1.26 (0.44) | 1.24 (0.47) | 1.25 (0.44) | 1.22 (0.42) | 1.24 (0.47) | 1.23 (0.45) | 1.21 (0.42) | 1.23 (0.48) | 1.2 (0.46) | 1.22 (0.47) | 1.2 (0.45) | 1.3 (0.5) | 1.19 (0.39) | 1.19 (0.45) |
| WBC (1000/μL) | 10.79 (6.24) | 10.59 (6.05) | 10.85 (6.21) | 10.81 (6.19) | 10.53 (5.98) | 13.34 (7.29) | 12.16 (6.86) | 10.82 (5.75) | 10.16 (5.07) | 10.75 (5.78) | 9.8 (4.87) | 11.61 (6.49) | 8.59 (4.94) | 9.91 (4.77) |
| RBC (million/mm^3^) | 4.11 (0.86) | 4.09 (0.83) | 4.13 (0.86) | 4.14 (0.83) | 4.09 (0.83) | 4.13 (0.83) | 4.13 (0.83) | 4.11 (0.81) | 4.19 (0.76) | 4.1 (0.82) | 4.16 (0.78) | 3.95 (0.82) | 4.38 (0.75) | 4.26 (0.73) |
| Platelets (1000/mm^3^) | 227.85 (113.44) | 234.28 (113.03) | 229.08 (116.09) | 235.36 (116.98) | 234.51 (112.01) | 253.46 (132.96) | 250.81 (128.19) | 238.32 (111.45) | 244.29 (106.69) | 240.3 (114.61) | 251.35 (110.62) | 230.43 (123.69) | 226.65 (98.74) | 248.29 (102.75) |
| Neutrophils (%) | 78.04 (11.91) | 76.28 (12.21) | 78.24 (11.93) | 76.29 (12.54) | 76.07 (12.29) | 79.89 (12.7) | 77.5 (13.07) | 76.27 (12.96) | 73.77 (13.02) | 75.88 (12.95) | 72.84 (12.68) | 78.67 (13.48) | 75.45 (12.46) | 73.01 (13.15) |
| Lymphocytes (%) | 12.03 (9.29) | 13.19 (9.65) | 11.78 (9.17) | 13.02 (9.89) | 13.33 (9.71) | 11.51 (11.56) | 12.96 (11.5) | 14.25 (10.39) | 16.89 (10.99) | 14.47 (10.54) | 17.47 (10.88) | 11.5 (10.19) | 15.2 (9.97) | 18.62 (11.27) |
| Monocytes (%) | 6.52 (3.29) | 6.58 (3.27) | 6.57 (3.41) | 6.68 (3.44) | 6.59 (3.24) | 6.03 (3.31) | 6.07 (3.26) | 6.78 (3.35) | 7.14 (3.34) | 6.83 (3.28) | 7.16 (3.34) | 6.06 (3.4) | 7.15 (3.78) | 7.23 (3.23) |
| Hematocrit (%) | 37.0 (7.51) | 37.14 (7.25) | 37.02 (7.63) | 37.38 (7.39) | 37.09 (7.23) | 36.99 (7.33) | 37.16 (7.26) | 36.84 (7.17) | 37.5 (6.73) | 36.79 (7.26) | 37.31 (6.83) | 36.6 (7.22) | 38.69 (6.22) | 37.86 (6.43) |
| Lactate (mmol/L) | 1.48 (0.54) | 1.47 (0.54) | 1.48 (0.54) | 1.47 (0.54) | 1.47 (0.53) | 1.63 (0.56) | 1.6 (0.57) | 1.54 (0.56) | 1.5 (0.55) | 1.53 (0.56) | 1.47 (0.55) | 1.7 (0.58) | 1.43 (0.51) | 1.49 (0.55) |
| pH | 7.37 (0.1) | 7.37 (0.09) | 7.38 (0.09) | 7.38 (0.09) | 7.37 (0.09) | 7.38 (0.12) | 7.38 (0.12) | 7.38 (0.09) | 7.39 (0.09) | 7.38 (0.09) | 7.38 (0.11) | 7.34 (0.12) | 7.42 (0.08) | 7.39 (0.09) |
| AST (U/L) | 28.26 (9.99) | 27.73 (9.68) | 28.84 (10.39) | 27.65 (9.88) | 27.67 (9.59) | 27.66 (9.69) | 27.4 (9.35) | 26.91 (10.6) | 25.45 (10.0) | 27.29 (10.18) | 25.37 (9.6) | 29.95 (10.28) | 30.44 (10.23) | 24.71 (9.96) |
| ALT (U/L) | 29.19 (19.46) | 26.86 (18.0) | 30.61 (19.25) | 28.2 (18.19) | 26.85 (18.28) | 27.6 (18.67) | 26.06 (18.15) | 28.02 (18.52) | 26.36 (17.47) | 27.28 (17.8) | 24.9 (16.66) | 30.06 (21.47) | 32.09 (19.79) | 26.39 (17.33) |
| SIRS | 1.64 (0.62) | 1.6 (0.61) | 1.66 (0.63) | 1.63 (0.63) | 1.59 (0.61) | 1.76 (0.67) | 1.67 (0.67) | 1.5 (0.57) | 1.39 (0.53) | 1.48 (0.57) | 1.37 (0.54) | 1.75 (0.71) | 1.49 (0.6) | 1.36 (0.52) |
| Male | 393 | 616 | 317 | 449 | 643 | 779 | 1030 | 4010 | 13000 | 2479 | 4750 | 438 | 1197 | 17941 |
| Female | 328 | 549 | 245 | 371 | 574 | 595 | 809 | 4143 | 14415 | 2472 | 5033 | 340 | 1002 | 22281 |

Footnotes: (1) International Normalized Ratio

**Table S3.** Descriptive statistics for test dataset. The values were obtained using the overall average and standard deviation of all times series values for all patients. The values for male and female variables are counts. The ALL column is the overall feature mean(std) or count for test data. Note that the sets of patients in each target are not necessarily mutually exclusive.

|  | ARDS_1 | ARDS_2 | ARDS_3 | ARDS_4 | ARDS_5 | Sepsis_6 | Sepsis_7 | Hypoxemia_8 | Hypoxemia_9 | Hypoxemia_10 | Hypoxemia_11 | Death | Covid19 | ALL |
| --- | --- | --- | --- | --- | --- | --- | --- | --- | --- | --- | --- | --- | --- | --- |
| Age (years) | 63.36 (16.3) | 65.21 (16.64) | 62.98 (16.51) | 65.06 (17.02) | 65.43 (16.56) | 67.82 (12.86) | 67.82 (12.86) | 67.7 (14.94) | 67.25 (15.17) | 66.85 (15.43) | 67.31 (15.28) | 77.0 (14.29) | 66.64 (16.3) | 65.4 (16.45) |
| SystolicBP (mmHg) | 129.64 (23.8) | 130.78 (23.66) | 128.95 (23.41) | 130.44 (23.56) | 130.37 (23.62) | 129.87 (25.61) | 129.87 (25.61) | 131.91 (24.51) | 132.72 (24.12) | 132.84 (25.05) | 134.64 (24.69) | 120.28 (27.27) | 131.23 (23.33) | 132.73 (23.91) |
| DiastolicBP (mmHg) | 70.53 (11.5) | 70.65 (11.48) | 70.52 (11.76) | 70.95 (11.62) | 70.53 (11.4) | 73.13 (13.46) | 73.13 (13.46) | 72.52 (11.94) | 74.06 (12.33) | 73.62 (11.95) | 75.07 (12.49) | 60.02 (11.18) | 72.17 (12.3) | 74.5 (12.21) |
| HR (BPM) | 91.71 (14.21) | 90.01 (15.62) | 91.91 (14.51) | 90.63 (15.66) | 90.05 (15.47) | 87.6 (16.8) | 87.6 (16.8) | 85.05 (16.39) | 83.93 (16.9) | 84.94 (17.17) | 82.5 (17.34) | 81.15 (15.8) | 86.08 (16.57) | 83.93 (17.44) |
| Temp (ºC) | 37.45 (1.18) | 37.35 (1.12) | 37.43 (1.17) | 37.33 (1.13) | 37.37 (1.13) | 37.69 (1.46) | 37.69 (1.46) | 37.06 (0.89) | 36.93 (0.71) | 37.05 (0.87) | 36.81 (0.58) | 37.57 (1.17) | 37.31 (0.93) | 36.89 (0.67) |
| RespRate (BPM) | 23.57 (5.67) | 23.19 (5.46) | 23.67 (5.74) | 23.17 (5.53) | 23.3 (5.47) | 24.98 (5.48) | 24.98 (5.48) | 20.74 (5.1) | 19.53 (4.34) | 20.2 (5.15) | 18.91 (3.74) | 22.03 (7.44) | 21.06 (5.12) | 19.26 (4.16) |
| SpO2 (%) | 93.11 (4.73) | 93.82 (4.74) | 93.15 (4.83) | 93.76 (4.78) | 93.93 (4.76) | 94.77 (5.4) | 94.77 (5.4) | 94.34 (3.85) | 95.73 (2.97) | 95.73 (2.34) | 97.77 (1.32) | 95.67 (4.1) | 95.26 (3.7) | 96.22 (2.89) |
| PaO2 (mmHg) | 74.42 (22.89) | 74.64 (22.52) | 75.17 (23.36) | 76.01 (23.21) | 74.64 (22.52) | 95.12 (33.35) | 95.12 (33.35) | 83.22 (24.78) | 86.0 (22.96) | 84.72 (22.48) | 92.86 (22.75) | 65.28 (6.89) | 73.54 (20.44) | 88.03 (23.23) |
| Creatinine (mg/dL) | 1.04 (0.4) | 1.04 (0.41) | 1.07 (0.4) | 1.07 (0.4) | 1.04 (0.4) | 1.26 (0.52) | 1.26 (0.52) | 1.05 (0.55) | 1.02 (0.52) | 1.06 (0.55) | 1.04 (0.54) | 1.15 (0.58) | 1.05 (0.5) | 1.01 (0.51) |
| BUN (mg/dL) | 22.05 (9.94) | 21.84 (9.95) | 22.29 (10.14) | 22.33 (10.04) | 22.09 (10.0) | 22.21 (11.71) | 22.21 (11.71) | 22.03 (10.64) | 20.45 (10.11) | 21.76 (10.17) | 20.15 (10.42) | 27.17 (12.84) | 21.07 (10.51) | 19.72 (10.09) |
| Bilirubin (mg/dL) | 0.56 (0.37) | 0.54 (0.34) | 0.55 (0.37) | 0.53 (0.35) | 0.54 (0.34) | 0.59 (0.38) | 0.59 (0.38) | 0.61 (0.52) | 0.62 (0.57) | 0.65 (0.54) | 0.6 (0.52) | 0.61 (0.44) | 0.6 (0.5) | 0.61 (0.57) |
| Glucose (mg/dL) | 139.63 (64.69) | 134.25 (60.72) | 137.48 (65.53) | 132.44 (61.75) | 133.48 (60.36) | 139.29 (42.06) | 139.29 (42.06) | 135.48 (80.62) | 137.63 (75.66) | 138.92 (89.28) | 138.11 (80.65) | 141.08 (43.99) | 143.4 (83.51) | 137.67 (78.06) |
| INR ^1^ | 1.17 (0.15) | 1.19 (0.16) | 1.18 (0.14) | 1.19 (0.16) | 1.21 (0.21) | 1.61 (0.96) | 1.61 (0.96) | 1.27 (0.59) | 1.23 (0.52) | 1.19 (0.42) | 1.21 (0.5) | 1.62 (1.07) | 1.29 (0.56) | 1.22 (0.5) |
| WBC (1000/μL) | 8.09 (3.51) | 8.62 (3.79) | 8.11 (3.57) | 8.58 (3.83) | 8.63 (3.75) | 8.35 (4.7) | 8.35 (4.7) | 8.97 (3.99) | 9.18 (4.09) | 8.82 (3.7) | 9.17 (3.92) | 7.55 (2.8) | 8.22 (3.45) | 9.14 (4.17) |
| RBC (million/mm^3^) | 4.38 (0.88) | 4.35 (0.81) | 4.41 (0.89) | 4.39 (0.82) | 4.37 (0.81) | 4.05 (0.7) | 4.05 (0.7) | 4.1 (0.84) | 4.1 (0.79) | 4.09 (0.86) | 4.0 (0.76) | 3.86 (0.65) | 4.33 (0.75) | 4.09 (0.79) |
| Platelets (1000/mm^3^) | 238.05 (136.93) | 240.1 (134.98) | 243.72 (137.61) | 244.59 (136.79) | 241.13 (133.88) | 249.67 (130.89) | 249.67 (130.89) | 233.17 (102.01) | 229.89 (94.58) | 225.91 (96.21) | 228.57 (94.63) | 180.04 (62.14) | 233.51 (97.33) | 233.73 (99.11) |
| Neutrophils (%) | 77.25 (10.73) | 77.09 (10.4) | 77.39 (10.85) | 77.24 (10.6) | 77.28 (10.4) | 76.04 (10.53) | 76.04 (10.53) | 75.99 (11.28) | 73.17 (11.52) | 75.6 (10.48) | 72.06 (11.44) | 78.81 (13.03) | 76.47 (10.53) | 72.1 (11.93) |
| Lymphocytes (%) | 14.17 (8.57) | 14.2 (8.1) | 14.0 (8.68) | 14.05 (8.26) | 14.09 (8.06) | 14.53 (9.17) | 14.53 (9.17) | 13.74 (8.25) | 16.05 (9.63) | 14.01 (7.93) | 16.8 (10.04) | 10.94 (7.08) | 14.14 (8.23) | 17.12 (10.16) |
| Monocytes (%) | 7.25 (3.07) | 7.34 (3.11) | 7.27 (3.11) | 7.38 (3.18) | 7.29 (3.1) | 7.97 (2.59) | 7.97 (2.59) | 7.91 (3.91) | 8.19 (3.31) | 7.8 (2.98) | 8.29 (2.92) | 8.68 (7.42) | 7.84 (3.96) | 8.19 (3.32) |
| Hematocrit (%) | 38.11 (7.28) | 38.07 (6.87) | 38.15 (7.45) | 38.05 (7.06) | 38.26 (6.93) | 34.65 (6.79) | 34.65 (6.79) | 37.02 (6.85) | 37.38 (6.47) | 36.95 (7.27) | 36.65 (6.45) | 34.46 (6.09) | 38.5 (6.22) | 37.33 (6.46) |
| Lactate (mmol/L) | 1.51 (0.43) | 1.5 (0.43) | 1.53 (0.42) | 1.51 (0.43) | 1.5 (0.42) | 1.59 (0.46) | 1.59 (0.46) | 1.44 (0.57) | 1.41 (0.55) | 1.44 (0.55) | 1.42 (0.49) | 1.53 (0.43) | 1.4 (0.48) | 1.41 (0.54) |
| pH | 7.4 (0.08) | 7.39 (0.08) | 7.4 (0.08) | 7.4 (0.08) | 7.39 (0.08) | 7.3 (0.19) | 7.3 (0.19) | 7.38 (0.08) | 7.37 (0.08) | 7.39 (0.07) | 7.37 (0.07) | 7.4 (0.1) | 7.39 (0.08) | 7.37 (0.09) |
| AST (U/L) | 31.14 (11.87) | 30.95 (11.36) | 31.63 (11.81) | 31.34 (11.29) | 30.87 (11.21) | 20.76 (11.53) | 20.76 (11.53) | 25.91 (10.94) | 24.97 (10.52) | 25.17 (10.93) | 23.45 (10.22) | 28.74 (10.62) | 28.63 (10.89) | 24.64 (10.47) |
| ALT (U/L) | 34.68 (17.0) | 34.41 (17.09) | 35.88 (16.59) | 35.41 (16.77) | 34.4 (16.9) | 31.63 (16.57) | 31.63 (16.57) | 29.6 (18.57) | 27.79 (18.74) | 27.59 (16.82) | 24.48 (17.32) | 25.38 (12.2) | 32.76 (17.71) | 27.49 (17.97) |
| SIRS | 1.81 (1.01) | 1.72 (0.98) | 1.8 (1.03) | 1.71 (1.0) | 1.72 (0.98) | 1.98 (1.2) | 1.98 (1.2) | 1.58 (0.78) | 1.53 (0.74) | 1.67 (0.86) | 1.42 (0.63) | 1.21 (0.39) | 1.67 (0.8) | 1.5 (0.72) |
| Male | 27 | 32 | 27 | 32 | 33 | 16 | 16 | 140 | 405 | 101 | 199 | 10 | 132 | 500 |
| Female | 17 | 20 | 15 | 17 | 20 | 6 | 6 | 117 | 361 | 74 | 165 | 15 | 96 | 459 |

Footnotes: (1) International Normalized Ratio


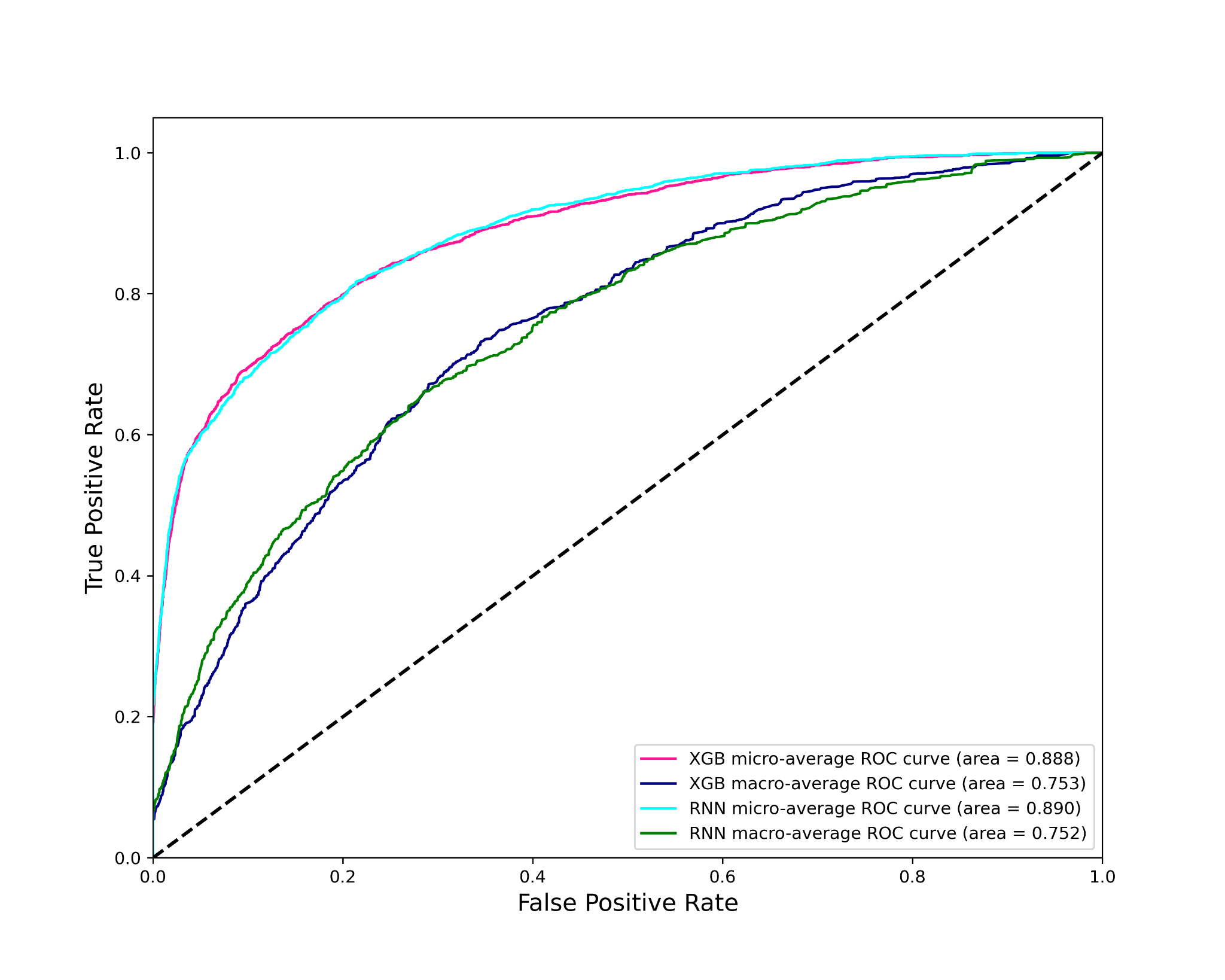


**Figure S1.** Micro and Macro-average ROC curve for XGB and RNN-13 models. The dashed line represents a baseline model. RNN, Recurrent Neural Networks; ROC, Receiver Operating Characteristic; XGB, XGBoost. Micro-averaging calculates metrics globally by considering each element of the label indicator matrix as a label, whereas macro-averaging calculates metrics for each label, and finds their unweighted mean.

**Table S4.** Distribution of the continuous input features in each of the three clusters of the embeddings of the first fully connected layer of the model. The values are given as mean (standard deviation).

| **Cluster** | **A** | **B** | **C** |
| --- | --- | --- | --- |
| **Age (years)** | 64.82 (14.91) | 64.99 (14.45) | 62.43 (16.51) |
| **SystolicBP (mmHg)** | 123.61 (23.97) | 130.27 (24.15) | 129.85 (23.08) |
| **DiastolicBP (mmHg)** | 70.51 (13.56) | 73.23 (13.43) | 72.43 (12.70) |
| **HR (BPM)** | 92.60 (19.64) | 85.71 (19.37) | 82.81 (18.62) |
| **Temp (ºC)** | 37.02 (0.78) | 36.87 (0.61) | 36.73 (0.44) |
| **RespRate (BPM)** | 21.40 (5.07) | 19.17 (3.70) | 18.45 (3.51) |
| **SpO2 (%)** | 93.53 (4.03) | 97.64 (1.67) | 95.66 (2.46) |
| **Creatinine (mg/dL)** | 1.17 (0.55) | 1.11 (0.51) | 1.05 (0.52) |
| **BUN (mg/dL)** | 23.16 (11.16) | 24.16 (11.49) | 19.99 (9.79) |
| **Bilirubin (mg/dL)** | 0.80 (0.65) | 0.71 (0.51) | 0.68 (0.47) |
| **Glucose (mg/dL)** | 152.53 (69.90) | 150.51 (96.65) | 148.61 (72.10) |
| **INR** | 1.26 (0.41) | 1.21 (0.46) | 1.26 (0.57) |
| **WBC (1000/μL)** | 10.66 (6.12) | 10.44 (5.82) | 9.79 (5.79) |
| **RBC** | 4.16 (0.86) | 3.97 (0.80) | 4.09 (0.79) |
| **Platelets (1000/μL)** | 227.00 (109.58) | 233.21 (107.30) | 234.91 (113.65) |
| **Neutrophils (%)** | 80.11 (11.14) | 75.27 (12.17) | 72.11 (11.05) |
| **Lymphocytes (%)** | 10.96 (9.22) | 14.20 (9.44) | 14.55 (9.67) |
| **Monocytes (%)** | 6.21 (3.30) | 6.80 (3.20) | 6.61 (3.66) |
| **Hematocrit (%)** | 37.41 (7.52) | 36.26 (6.93) | 36.74 (7.19) |
| **Lactate (mmol/L)** | 1.48 (0.54) | 1.46 (0.54) | 1.35 (0.50) |
| **pH** | 7.38 (0.10) | 7.39 (0.10) | 7.38 (0.07) |
| **AST (1/L)** | 29.48 (10.00) | 27.64 (9.23) | 24.40 (9.38) |
| **ALT (1/L)** | 32.65 (21.25) | 25.28 (16.93) | 22.11 (13.53) |
| **SIRS** | 1.51 (1.01) | 1.05 (0.94) | 0.86 (0.86) |

**A.**


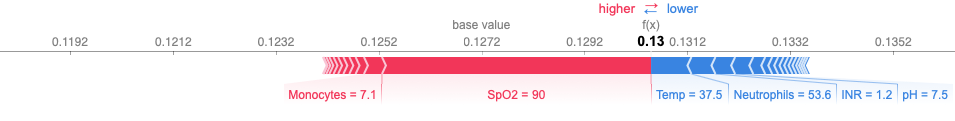


**B.**


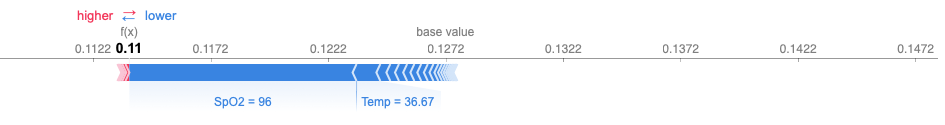


**C.**


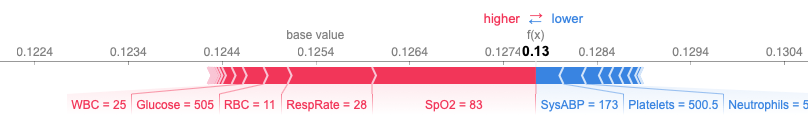


**D.**


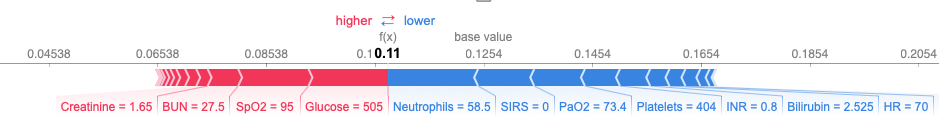


**Figure S2**. Force plot for 4 different patients. **(A)** represents an ARDS patient who is predicted by the Recurrent Neural Networks (RNN) as having ARDS (True Positive, TP). **(B)** represents a non-ARDS patient who is predicted by the RNN as not having ARDS (True Negative, TN). **(C)** represents an non-ARDS patient who is predicted by the RNN as having ARDS (False Positive, FP). **(D)** represents an ARDS patient who is predicted by the RNN as not having ARDS (False Negative, FN). Note: We use ARDS-5* definition from **Table 3.**

**Table S5.** Comparison of performances of the RNN13 models with and without the attention layer on each of the outcomes. The table shows the AUROC of each model for each of the labels. We note that the RNN13 model with the attention layer performs better than that without the attention layer model on 10/13 outputs. AUROC, Area Under the Receiver Operating Characteristic curve; CI, Confidence Interval; RNN, Recurrent Neural Network.

| Labels | RNN13 without Attention | RNN13 with Attention |
| --- | --- | --- |
|  | AUROC  (95% CI) | AUROC  (95% CI) |
| ARDS-1 | 0.740 (0.671 - 0.805) | 0.842 (0.794 - 0.888) |
| ARDS-2 | 0.703 (0.625 - 0.779) | 0.791 (0.746 - 0.836) |
| ARDS-3 | 0.729 (0.663 - 0.796) | 0.845 (0.795 - 0.894) |
| ARDS-4 | 0.695 (0.619 - 0.769) | 0.812 (0.768 - 0.858) |
| ARDS-5 | 0.698 (0.624 - 0.775) | 0.795 (0.751 - 0.839) |
| Sepsis-6 | 0.517 (0.386 - 0.657) | 0.626 (0.533 - 0.714) |
| Sepsis-7 | 0.498 (0.363 - 0.641) | 0.586 (0.481 - 0.681) |
| Hypoxemia-8 | 0.736 (0.701 - 0.772) | 0.739 (0.708 - 0.77) |
| Hypoxemia-9 | 0.837 (0.809 - 0.863) | 0.834 (0.81 - 0.855) |
| Hypoxemia-10 | 0.646 (0.607 - 0.688) | 0.638 (0.601 - 0.673) |
| Hypoxemia-11 | 0.881 (0.860 - 0.9) | 0.880 (0.862 - 0.897) |
| Death-12 | 0.607 (0.497 - 0.717) | 0.700 (0.625 - 0.768) |
| Covid-13 | 0.620 (0.580 - 0.664) | 0.673 (0.637 - 0.714) |

**Table S6.** Grid search hyperparameters.

| **Model** | **Parameter** | **Values** |
| --- | --- | --- |
| RNN | num_epochs | [4, 5] |
|  | batch_size | 256 |
|  | L2_reg | [1e-10, 1e-8] |
|  | max_norm | 1.0 |
|  | learning_rate | [0.002, 0.001] |
|  | gamma | 0.5 |
|  | patience | 1 |
| XGB | max_depth | [3, 7] |
|  | learning_rate | [0.05, 0.1] |
|  | reg_lambda | [0.5, 1.0] |
|  | scale_pos_weight | [1, 3] |
|  | n_estimators | [50, 100] |
